# Supplementary material for: Causal Relationship between Aging and Anorexia Nervosa: A White-Matter-Microstructure-Mediated Mendelian Randomization Analysis
Source: Biomedicines. 2024 Aug 16;12(8):1874. doi: 10.3390/biomedicines12081874 (PMC11351342; doi:10.3390/biomedicines12081874)
Supplement: Supplementary file 1 [file biomedicines-12-01874-s001.zip › Supplementary Figure/Figure S4/Singe SNP plot of MR analysis for TL to ubm-b-1681.pdf]

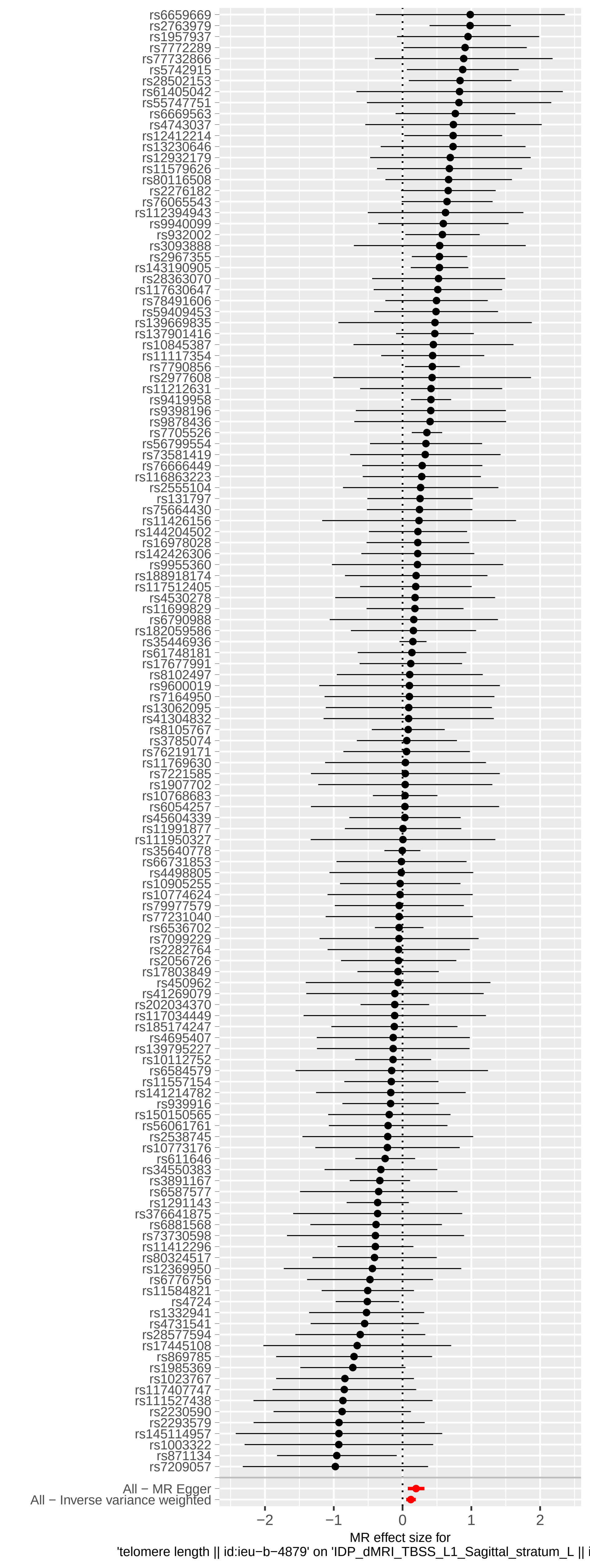
rs6659669
rs2763979
rs1957937
rs7772289
rs77732866
rs5742915
rs28502153
rs61405042
rs55747751
rs6669563
rs4743037
rs12412214
rs13230646
rs12932179
rs11579626
rs80116508
rs2276182
rs76065543
rs112394943
rs9940099
rs932002
rs3093888
rs2967355
rs143190905
rs28363070
rs117630647
rs78491606
rs59409453
rs139669835
rs137901416
rs10845387
rs11117354
rs7790856
rs2977608
rs11212631
rs9419958
rs9398196
rs9878436
rs7705526
rs56799554
rs73581419
rs76666449
rs116863223
rs2555104
rs131797
rs75664430
rs11426156
rs144204502
rs16978028
rs142426306
rs9955360
rs188918174
rs117512405
rs4530278
rs11699829
rs6790988
rs182059586
rs35446936
rs61748181
rs17677991
rs8102497
rs9600019
rs7164950
rs13062095
rs41304832
rs8105767
rs3785074
rs76219171
rs11769630
rs7221585
rs1907702
rs10768683
rs6054257
rs45604339
rs11991877
rs111950327
rs35640778
rs66731853
rs4498805
rs10905255
rs10774624
rs79977579
rs77231040
rs6536702
rs7099229
rs2282764
rs2056726
rs17803849
rs450962
rs41269079
rs202034370
rs117034449
rs185174247
rs4695407
rs139795227
rs10112752
rs6584579
rs11557154
rs141214782
rs939916
rs150150565
rs56061761
rs2538745
rs10773176
rs611646
rs34550383
rs3891167
rs6587577
rs1291143
rs376641875
rs6881568
rs73730598
rs11412296
rs80324517
rs12369950
rs6776756
rs11584821
rs4724
rs1332941
rs4731541
rs28577594
rs17445108
rs869785
rs1985369
rs1023767
rs117407747
rs111527438
rs2230590
rs2293579
rs145114957
rs1003322
rs871134
rs7209057
All − MR Egger
All − Inverse variance weighted
−2
−1
0
1
2
MR effect size for
'telomere length || id:ieu−b−4879' on 'IDP_dMRI_TBSS_L1_Sagittal_stratum_L || i
